# Effectiveness of expanded prenatal screening among consanguineous couples of Afghan descent

**DOI:** 10.1016/j.xagr.2025.100503

**Published:** 2025-05-05

**Authors:** Noura Osman, Laila Rhee, Nina Boe, Herman Hedriana, Krishna Singh

**Affiliations:** 1 UC Davis Health Prenatal Diagnosis of Northern California, Sacramento, CA (Osman, Rhee, Boe, Hedriana, and Singh); 2 Department of Obstetrics and Gynecology, Division of Maternal Fetal Medicine, UC Davis Health, Sacramento, CA (Hedriana and Singh)

**Keywords:** single gene disorders, genetic counseling, sociological, prenatal diagnosis, delivery of care

## Abstract

**Objective:**

The American College of Medical Genetics recommends extensive carrier screening among consanguineous couples, yet limited information is available regarding its performance among specific populations. We describe the potential utility of a large expanded carrier screening panel (>500 genes) for Afghan couples with consanguinity.

**Method:**

A retrospective chart review was conducted of all patients who reported consanguinity and had genetic counseling consultation between 2010 and 2021 at our institution.

**Results:**

Thirty-six women of Afghan descent reported consanguinity. Nineteen (53%) did not have known autosomal recessive disease risk (no prior fetus/child with suspected syndrome), 11 (31%) had apparent disease risk (fetus/child with symptoms suggesting syndrome but no specific diagnosis), and six (17%) had known molecular diagnosis for a prior child with a recessive syndrome. Of the six women with known molecular diagnosis for prior child with syndrome, five had known pathogenic variants confirming a genetic disorder, and one had highly suspicious variant of uncertain significance in the family. Among these six, five (83%) could have been identified with a 787-gene prenatal carrier screening panel.

**Conclusion:**

Large, expanded carrier screening panel appears to be an effective method for identifying disorders among consanguineous Afghan couples and should ideally be considered preconceptionally.

**AJOG Global Reports at a Glance**

**Why was this study conducted?**

To assess the yield of large prenatal carrier screening panel in consanguineous couples of Afghan ancestry.

**Key findings**

Our study suggests that readily available expanded prenatal carrier screens help to reveal disease-risk in consanguineous couples of Afghan ancestry.

**What does this add to what is known?**

Our study provides additional data highlighting that large carrier screening panels provide useful disease-risk information in a high-risk population that has previously not been well studied.

## Introduction

Consanguinity is a well-established concept in the medical-genetic literature that is generally used to describe unions between two people who are second cousins or closer.^1^ Consanguineous unions are driven by a variety of geographic, economic, social, religious and/or cultural factors and are highly prevalent in areas of the Middle East, Asia, and Africa.^2^ Of note, it is estimated that 46% of unions in Afghanistan are consanguineous, with the majority being between first cousins.^3^ In the last decade, the increasing instability, economic hardship, and restricted access to education and health care in Afghanistan has led to an influx of individuals seeking refuge in other countries.^4^ Specifically, as of 2022, greater than 100,000 Afghan refugees were living in the United States, with about 12,000 in California, including approximately 9700 in the Greater Sacramento region.^5^ Although an increased risk of autosomal recessive (AR) conditions among offspring of consanguineous unions is not new, this knowledge is particularly pertinent in those of Afghan ancestry. For example, the reported prevalence of metabolic disorders alone (without other heritable conditions) is estimated at 39% among newborns in this population compared to a worldwide estimate of 0.1%.^4^

The American College of Medical Genetics (ACMG) has recommended more extensive carrier screening among consanguineous couples.^6^ While the aim is to offer carrier screening that is more inclusive of diverse populations, it must be noted that knowledge of the clinical utility of carrier screening is well established in Ashkenazi Jewish, North American and European populations, but few studies have been published about the Afghan population.^6^ In this study, we describe the potential utility of a large, expanded carrier screening panel (>500 genes) for Afghan couples with consanguinity.

## Methods

A retrospective chart review was performed of all Afghan patients referred for genetic counseling at the University of California Davis Health Prenatal Diagnosis Center between 2010 and 2021 who reported consanguinity. The study is deemed exempt by the UC Davis Institutional Research Board (IRB #1889130). Patients were seen preconceptionally and/or during pregnancy. Indications for genetic counseling included prior pregnancy with fetal anomaly or demise, known family history of genetic disorder, consanguinity, advanced maternal age, abnormal fetal ultrasound, or abnormal prenatal/maternal serum screening result. Those who did not report Afghan descent were excluded. Descriptive data was collected and analyzed with Excel (Microsoft, Redmond, WA). During the study period, there was a steady increase in the number of AR conditions that could be detected and identified using the available expanded carrier screening. However, in our study, we used panels ranging from 101 to 421 genes, as panels with greater than 500 genes only became widely available after this study was completed.

## Results

By pedigree analysis and/or verbal patient report, 36 unrelated women of Afghan ancestry were identified to be in consanguineous unions. The partners included 21 (58%) first cousins, 7 (19%) second cousins, 3 (8%) first cousins once removed, 2 (6%) double first cousins once removed, 1 (3%) half-first cousins, 1 (3%) second cousins once removed, and 1 (3%) first cousins *and* second cousins (Table).

**Table:** Clinical and molecular data for consanguineous Afghan couples referred to prenatal/preconception genetics

Referral indication	Other relevant history	Relationship	Testing conducted; shared variant (boldface)	Conducting laboratory	Familial disease would have been detectable on 613 or 787-gene panel? ✓ Yes, ✗ no
Couples with suspected or confirmative molecular findings					
Family history of argininosuccinic aciduria in 1 of 3 offspring; consanguinity		1st cousins	**Homozygous c.637C>T pathogenic variant in *ASL***	Baylor	✓ 613, ✓ 787
Family history of phalangeal joint abnormalities and arthrogryposis affecting all 4 limbs in 2 of 4 offspring; consanguinity	• Same features reported in patient’s nephew Both members of the couple silent alpha-thalassemia carriers	1st and 2nd cousins	**Trio WES homozygous likely pathogenic variant c.351-9T>C in *CHRNG*** (multiple pterygium syndrome) 613-gene CS (+), *following the study period* Silent alpha thalassemia Congenital secretory chloride diarrhea Multiple pterygium syndrome	GeneDx Natera	✓ 613, ✓ 787
Skeletal dysplasia identified on second trimester ultrasound	• History of two miscarriages	1st cousins	Cystic fibrosis **Skeletal dysplasia gene panel homozygous likely pathogenic variant c.337G>A in *DDR2*** (autosomal recessive spondylometaepiphyseal dysplasia)	Invitae	✗ 613, ✓ 787
Family history of cutis laxa type II (De Barsy syndrome) in one of three children; consanguinity		1st cousins	**Trio WES homozygous, likely pathogenic variant c.752G>A in *PYCR1*** A variant of unknown significance, c.214G>T, was identified in *L1CAM* 101-gene CS	Prevention genetics Myriad	✗ 613, ✓ 787
Family history of *TBCK-*intellectual disability syndrome in one of two children, consanguinity, and high-risk maternal serum screen for trisomy 21 and SLOS, and ONTD	• Fetal hydrops identified on ultrasound; fetus deceased Same features of *TBCK-syndrome* reported in patient’s niece (parents reported nonconsanguineous)	1st cousins	**Fetal WES homozygous pathogenic variant c.18676 C>T in *NEB*** (*NEB*-related nemaline myopathy); negative for *TBCK* familial variant; typical CMA complement with 14% ROH	GeneDx	NEB ✗ 613, ✓ 787
Family History of two children who died of unspecified neurological disorder, consanguinity, Advanced maternal age	• Niece reported to be “confined to a wheelchair” Additional niece (different than above family) with same reported features as patient’s deceased children	Double 1st cousins once removed	**Homozygous VUS in *TSEN54* c. 636 G>T, p. K212N.** 274-gene CS (–) *Muscle eye brain disease*	GeneDx Natera	✓ 613, ✓ 787
Couples without suspected or confirmative molecular findings based on carrier screening					
Family history of unspecified hypotrichosis syndrome in 3 of 4 offspring; consanguinity	• Same reported features in patient’s nephew and cousins History of intrauterine fetal demise	1st cousins	274-gene CS (–)	Natera	Causative gene not identified. CS panels include hypotrichosis 8, short stature, onychodysplasia, facial dysmorphism, and hypotrichosis syndrome, cartilage-hair hypoplasia, cartilage-hair hypoplasia anauxetic dysplasia spectrum disorder
Family history of IUFD		1st cousins	274-gene CS (–)	Natera	
Family history of autism in daughter	• One miscarriage • Reported autism in three nephews and two nieces	1st cousins	274-gene CS (–)	Natera	
Consanguinity		1st cousins	176-gene CS (–)	Myriad	
High-risk trisomy 21 on cell-free DNA screening; history of transposition of great arteries in 1 of 3 offspring	• One miscarriage • History of cryptorchidism and knee hemangioma in second son	1st cousins	421-gene CS (+) *Carnitine deficiency* *Congenital adrenal hyperplasia, 21-hydroxylase deficiency* *Mucopolysaccharidosis, Type 1 (Hurler syndrome)*	Natera	
IUGR on US; consanguinity	• Reported 6 healthy children	1st cousins	274-gene CS (+) *Biotinidase deficiency*	Natera	
NND in daughter at 4.5 mo of life following chronic respiratory distress episodes; consanguinity	• Reported 1 other healthy child	1st cousins	274-gene CS (+) *Inclusion body myopathy 2*; partner negative	Natera	
Consanguinity	• Reported 4 healthy children	1st cousins once removed	CF and SMA only; declined additional CS	Quest	
Recurrent pregnancy loss; consanguinity		1st cousins	421-gene CS (–)	Natera	
History of neonatal demise		1st cousins once removed	274-gene CS (–)	Natera	
Recurrent pregnancy loss, consanguinity, and advanced maternal age		Double 1st cousins once removed	176-gene CS (+) *Silent alpha-thalassemia* *Spinal muscular atrophy*	Myriad	
Consanguinity		1st cousins	421-gene CS (+) *Inclusion body myopathy 2*	Natera	
Fetal US findings of increased nuchal fold and abnormal cranium shape	• Reported 2 nieces with developmental regression around 4 y of life • Reported 6 healthy children	Half 1st cousins	274-gene CS (–) *Biotinidase deficiency* *Cystic fibrosis* *Krabbe disease*	Natera	
Infertility		1st cousins	274-gene CS (–)	Natera	
Family history of West syndrome	• Reported history of IUFD, neonatal demise (11 mo of life) • Daughter with West syndrome had negative or nondiagnostic testing, which included a focused exome, chromosomal microarray, fragile X testing, and an epilepsy panel • Reported history of 2 healthy children	1st cousins once removed	274-gene CS (+) *Biotinidase deficiency* *Usher syndrome, type 2A*	Natera	
Recurrent pregnancy loss; consanguinity	• History of confirmed monosomy x in previous pregnancy loss • One (of two children) with reported autism	2nd cousins once removed	421-gene CS (+) *Leber congenital amaurosis 2*	Natera	
Family history of chromosomal translocation (47,XX,+der(22)t(11;22)(q23.3;q11.2))	• Two reported miscarriages • Two reported healthy children	2nd cousins	176-gene CS (+) *Congenital adrenal hyperplasia, CYP21A2-related* *Peroxisome biogenesis disorder type 3*	Myriad	
Couples not known to have undergone/declined carrier screening					
Family history of IUFD; consanguinity		1st cousins	Declined carrier exome due to cost	Declined	
CHD and situs inversus on US	Three healthy children	2nd cousins	Declined carrier screening		
Serum screening positive trisomy 18 or Smith–Lemli–Opitz syndrome; consanguinity		2nd cousins			
Advanced maternal age		2nd cousins			
Genetic carrier (beta thalassemia) and consanguinity	• Reported 16-wk IUFD Reported 3 healthy children	1st cousins	Did not complete expanded CS		
Consanguinity	• Reported 3 healthy children • Sister with reported history of two miscarriages • Half-first cousin with reported “child-like behavior” in adulthood • Cousin with reported neonatal demise	1st cousins		Declined CS	
Advanced maternal age		1st cousins	Declined carrier screening		
Family history of hearing loss and consanguinity		1st cousins		Declined CS	
Consanguinity	• Reported IUFD in first pregnancy Reported neonatal demise of unknown etiology in second child	2nd cousins		Declined CS	
Carrier of spinal muscular atrophy		2nd cousins		Declined CS	
History of neonatal demise of unknown etiology	• Two reported miscarriages • Reported two healthy children • Reported father of the baby’s sister whose *“brain is not growing”*	1st cousins			
Consanguinity		1st cousins			
Family history of father of the baby with situs inversus with heart valve replacement	• Reported multiple congenital anomalies and neonatal demise of unknown etiology in father of the baby’s brother • Reported another neonatal demise presumed infectious etiology in father of the baby’s second brother • Reported heart murmurs in father of the baby’s two sisters and nephew	2nd cousins			

*CHD*, congenital heart defect; *CMA*, chromosomal microarray; *CS*, carrier screening; *IUFD*, intrauterine fetal demise; *ROH*, regions of homozygosity; *SLOS/SCD, and ONTD*, Smith–Lemli–Opitz syndrome, and open neural tube defect; *US*, ultrasound; *WES*, whole-exome sequencing.

The majority (58%) of referral indications were for consanguinity with or without additional family history. Consanguinity was discovered incidentally during the genetic counseling session in 42% (Table). Twenty-three (64%) of the 36 women had no previous family history of a fetus or child with suspected syndrome, 11 (31%) had a previous fetus with suspected syndrome but without a specific diagnosis, and 6 (17%) had a previous fetus or child with a known AR molecular diagnosis (Figure).

**Figure:**
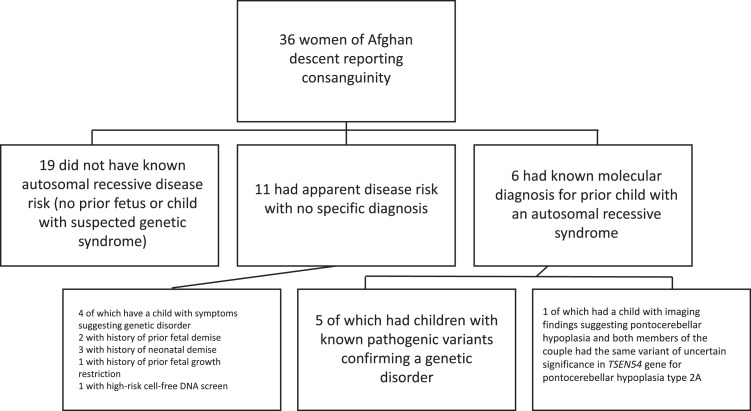
Summary of results

Osman. Effectiveness of expanded prenatal screening. AJOG Glob Rep 2025

Eighteen of the 36 couples (50%) did not undergo general carrier screening (not offered, did not complete, or declined), or carrier screening other than cystic fibrosis and/or spinal muscular atrophy. A total of 18 women underwent carrier screening for ≥101 genes. One underwent carrier screening for 101 genes, 3 for 176 genes, 10 for 274 genes, and 4 for 421 genes. None of the women in the current study period had an expanded carrier gene panel of more than 500 genes, as these panels only became widely available after this study period (Table). It is unknown whether the partners of these women also underwent carrier screening, when warranted. Of note, one couple with a previous history of a child affected with molecularly confirmed multiple pterygium syndrome (*CHRNG*) was seen for genetic counseling again in 2024. She elected to pursue 613-gene carrier screen, which identified the familial *CHRNG* mutation and her carrier status for alpha thalassemia and congenital secretory chloride diarrhea 1.

Of the 11 families without a specific diagnosis, four had a child with findings suggesting a genetic disorder: one with encephalopathy, one with heterotaxy, one with hypotrichosis, and one with intellectual disability (Table). In addition, two others had a history of prior fetal demise, three had history of prior neonatal demise, one had a history of fetal growth restriction (FGR), and one had a high-risk prenatal cell-free DNA screen. The latter 4 cases were also suggestive of a possible genetic disorder; however, no specific anomalies or genetic results were reported in the fetal/neonatal demise or FGR cases, and the patient with high-risk prenatal cell-free DNA screen did not have diagnostic genetic amniocentesis (Figure).

Of the six couples with prior progeny showing syndromic findings, five had known pathogenic variants confirming a genetic disorder, including arginosuccinic aciduria (*ASL* gene), Escobar syndrome (*CHRNG* gene), spondylometaphyseal dysplasia (*DDR2* gene), de Barsy progeria (*PYCR1* gene), and infantile hypotonia with psychomotor retardation and characteristic facies-3 (*TBCK* gene) (Table). The molecular etiologies of these cases were identified after previously affected children was evaluated by a Pediatric Geneticist. Whole-exome trio sequencing (WES) was conducted in the first cousin couple whose child had a *TBCK* variant due to a new ongoing pregnancy with FGR with hydrops. This revealed biallelic pathogenic variants in the *NEB* gene, resulting in a diagnosis of nemaline myopathy. One of the six couples had a deceased child with MRI findings consistent with pontocerebellar hypoplasia. Parental testing identified biallelic variants of uncertain significance (VUS) in the *TSEN54* gene (pontocerebellar hypoplasia type 2A), suggesting a causative etiology. All the above pathogenic variants, including *NEB*, could have been identified through an expanded 787-gene carrier screen. The *TSEN54* gene is also tested with the 787 panel, but it is unclear if the screening laboratory may have reported the specific variant that was identified. The *TBCK* variant is not included in the expanded 787-gene carrier screen (Figure).

## Discussion

To assess the potential clinical utility of expanded carrier screening in an understudied population, we evaluated 36 unrelated pregnant women of Afghan ancestry who were in consanguineous unions. Prior research has investigated WES among consanguineous couples with up to 56 percent yield for carrier condition match.^7^ However, to our knowledge, no studies have investigated the potential utility of readily available expanded carrier screens in individuals of Afghan ancestry. Although intuitively WES would provide a higher yield, it has limitations including higher cost, longer turnaround time, and a generally unfocused set of genes.^8^ More importantly, the clinical availability of WES in the context of consanguinity (eg, whole-exome carrier screening in a healthy individual) is scarce. In fact, from our own clinical experience, several labs have discontinued this testing over the past years (eg, MNG Laboratories, GeneDx). At the current time, expanded carrier screening panels are an available and feasible option for consanguineous families who are considering preconception and/or prenatal testing options. In this study, two panels are cited, the Fulgent Beacon (El Monte, CA) 787 gene panel and the Natera Horizon (Austin, TX) 613 gene panel, referred to henceforth as the 787 and 613 panels, respectively. In general, these panels include screening for AR and X-linked conditions, which have severe presentations, may present early in childhood, or by which early intervention may be beneficial.

In 2021, the ACMG published a comprehensive review with a goal of standardizing the practice of expanded carrier screening, with recommendations for a consistent and equitable approach for offering tiered carrier screening to those who are pregnant or considering pregnancy.^6^ ACMG recommends offering Tier 3 screening to all individuals who are pregnant or considering pregnancy. Tier 3 screening currently includes screening of 113 genes with a 1 in 200 carrier frequency. Both the 787 and the 613 panels include coverage of the 113 genes recommended for prenatal/preconception screening by the ACMG. Tier 4 screening, which supplements Tier 3, includes additional genes with carrier frequency of <1 in 200. Tier 4 screening should be considered when a pregnancy stems from a known or possible consanguineous relationship (defined as second cousins or closer), or when a personal/family history warrants. However, the ACMG does not specify the number of genes a Tier 4 panel should include at a minimum. Our study provides groundwork supporting the potential clinical utility of readily available larger expanded carrier screens in a significantly growing population with a high rate of consanguinity.

Pedigree analysis facilitated calculation of the coefficient of relationship in our study and revealed that most of the couples were in first-cousin unions. Most of the participants did not report a family history suggestive of AR predisposition (no history of previous fetus or child with a suspected syndrome); while a proportion of the couples had children with findings suspicious for underlying genetic etiology (encephalopathy, heterotaxy, hypotrichosis, intellectual disability, prior fetal demise, neonatal demise, FGR, and a high-risk prenatal screen) but without known workup. None of these participants had the 613 or 787 panel, as these were not available during the study period. However, the one family who presented for genetic counseling in a subsequent pregnancy following the study period did undergo the 613 panel, which identified the familial variant causative of multiple pterygium syndrome in their child. Thus, it is possible that AR predisposition might have been discovered in other cases with a 787 or 613 panel. In the couples who had a previous fetus or child with a known AR molecular diagnosis, five were due to likely and/or pathogenic variants, and one highly suspicious VUS. Tier 4 testing with the 787 or 613 panel would have identified 5 of 7 AR disease risks in our population, excluding the VUS in *TSEN54* (depending on lab variant classification) and the *TBCK* gene. Overall, 71% of cases with a known AR disorder in our study population could have potentially been identified preconceptionally with a larger expanded carrier screen.

It is important to note that consanguinity was only incidentally discovered during the genetic counseling in almost half of the cases. This implies that primary obstetric providers are not consistently addressing this history during prenatal care, although the information could be critical in guiding appropriate genetic counseling and testing. Asking patients about consanguinity during obstetric intake appointments would allow improved risk assessment and identify patients who should be referred for genetic counseling.

No large studies are available documenting the carrier spectrum among the Afghan population, therefore, more specific carrier screening guidelines are not currently in place for this group. Our study is limited by a relatively small cohort. Another limitation is the retrospective design, which included bias toward referral due to prior poor outcome (patients with a known pathogenic gene variant, prior affected child, or ultrasound-identified fetal anomalies might be preferentially referred to our tertiary care center for prenatal diagnostic testing, genetic counseling, and ultrasound). Many of our patients were seen during only one pregnancy, thus an added limitation, because even if they had an AR disease risk, they would have a 3 in 4 (75%) chance to have an unaffected offspring in any pregnancy. Lastly, because of the small cohort, our study is not large enough to identify specific genetic variations which may be occurring with higher frequency among Afghan couples. The strength of our study is the demonstration of the effectiveness of broader carrier screening for Afghans, which to our knowledge, has not been previously documented.

Larger prospective studies are needed to confirm the spectrum and frequency of genetic variants among Afghan couples to allow development of an optimal targeted carrier screening protocol. Given the reported prevalence of metabolic disorders estimated at 39% among newborns in the Afghan population,^4^ a larger, expanded carrier screen panel should be offered in regions where this population find their homes. Obstetric providers and any other providers providing prenatal or preconception care should ascertain a history of consanguinity at the initial visit and consider offering genetic counseling and the larger expanded carrier screen panel (eg, 787 panel) to couples of Afghan descent.

## CRediT authorship contribution statement

**Noura Osman:** Writing – review & editing, Writing – original draft. **Laila Rhee:** Writing – review & editing, Writing – original draft, Data curation. **Nina Boe:** Writing – review & editing, Writing – original draft. **Herman Hedriana:** Writing – review & editing, Supervision. **Krishna Singh:** Writing – review & editing, Writing – original draft, Supervision.
